# Exogenous oxytocin increases gaze to humans in male cats

**DOI:** 10.1038/s41598-024-59161-w

**Published:** 2024-04-18

**Authors:** Madoka Hattori, Kodzue Kinoshita, Atsuko Saito, Shinya Yamamoto

**Affiliations:** 1 Wildlife Research Center, Kyoto University, 2-24 Tanaka-Sekiden-cho, Sakyo-ku, Kyoto, 606-8203 Japan; 2 Graduate School of Asian and African Area Studies, Kyoto University, Research Bldg. No 2 Yoshida-Honmachi, Sakyo-ku, Kyoto, 606-8501 Japan; 3 Department of Psychology, Faculty of Human Sciences, Sophia University, 7-1 Kioicho, Chiyoda-ku, Tokyo, 102-8554 Japan; 4 Institute for Advanced Study, Kyoto University, Yoshida Ushinomiya-cho, Sakyo-ku, Kyoto, 606-8501 Japan

**Keywords:** Zoology, Animal behaviour, Animal physiology

## Abstract

Although oxytocin (OT) plays a role in bonding between heterospecifics and conspecifics, the effects of OT on the formation of such interspecific social behavior have only been investigated between humans and dogs (*Canis familiaris*). In this study, for comparative evaluation of the effects of OT between dog–human and cat–human social interaction, we investigated the effects of exogenous OT on the behavior of domestic cats (*Felis silvestris catus*) toward humans. We intranasally administered OT or saline to 30 cats using a nebulizer and recorded their behavior (gaze, touch, vocalization, and proximity). The results showed an interaction between the administration condition and sex for gaze duration. Post hoc analyses revealed a significant increase in gaze in the OT condition in male cats but not in females. There were no significant differences in gaze toward owners and strangers in any condition or sex. The male-specific OT-mediated increase in gaze toward humans observed in this study differs from previous research on dogs wherein such effects were observed only in females. These findings suggest an overall effect of exogenous OT on cats’ social relationship with humans as well as the possibility of different mechanisms between cat–human and dog–human relationships.

## Introduction

Oxytocin (OT) plays diverse roles in the social behavior of mammals and has recently garnered attention as a neurobiological foundation for bonding between individuals. OT primarily functions in reproductive processes, such as uterine contractions during childbirth and milk secretion, contributing to the formation of social bonds between mothers and offspring^1^. Furthermore, OT is associated with mother-offspring bonding, pair bonding, sexual behavior, and parental care^2^. However, the OT receptor system has been linked to differences in social behaviors depending on sex^3^, and there is variability across species^4^. Therefore, social behaviors involving OT should be examined in various species, including humans.

The role of OT in social behaviors in various mammalian species has been empirically investigated through OT administration. In humans, OT administration decreases the anxiety responses and cortisol secretion induced by psychological stress^5^. In prairie voles (*Microtus pennsylvanicus*), OT administration induces a partner preference for opposite-sex individuals^6^ but promotes aggression toward same-sex individuals^7^. In macaques (*Macaca mulatta*), OT administration increases the social interest of infants and their attachment behaviors toward their mothers^8^. In bonobos (*Pan paniscus*), OT administration increases eye contact and grooming between conspecifics^9,10^.

The OT nervous system has also been implicated in interspecific social relationships. Previous experiments with dogs have shown that exogenous OT promotes mutual gaze with the human owner^11^, indicating that OT-mediated increases in social behavior exist between different species. In the experiment on dogs (*Canis familiaris*), the intranasal administration of OT increased the gaze time of females toward their owners and simultaneously increased urinary OT on the owner’s side, although other behaviors, such as vocalization and proximity, did not change. This experiment showed that dog gazes toward their owners functioned as an attachment behavior and further promoted their owners' OT secretion, indicating an OT-mediated positive loop in bonding between dogs and humans. This study suggested that OT promotes social bonding between humans and companion animals.

Thus, the effects of OT on the formation of interspecific bonds and social relationship have been suggested; however, this has only been empirically investigated between dogs and humans. Cats (*Felis silvestris catus*), like dogs, have achieved companion animal status; however, whether they have the same social relationship with their owners as dogs is unknown. Wolves, the ancestral species of dogs, are pack animals, whereas African wild cats (*Felis silvestris lybica*), the ancestral species of domestic cats, are solitary animals^12^. In addition, companion animals have been domesticated for different lengths of time (cats, ~ 10,000 years^13^; dogs, ~ 15,000–100,000 years)^14,15^, and the domestication of cats is considered incomplete compared to that of dogs^16^. Nevertheless, studies observing attachment under experimental conditions (the Strange Situation Test) have shown that cats, like dogs, show attachment behaviors to humans^17,18^.

Remaining questions pertain to the similarities and differences between dogs and cats in terms of the mechanisms of the establishment of social relationships with their owners. The role of OT in social interaction between cats and their owners remains unknown, and the positive feedback loop between OT and eye gaze observed between dogs and their owners has not been identified in cats. Therefore, empirical studies involving the administration of exogenous OT in cats are essential but are currently lacking. Although there are no empirical studies on the effects of exogenous OT on cat–human interaction, a pilot study with four cats^19^ found that urinary OT increased with social contact with people who were not their owners but who cared for them, suggesting that human contact may be associated with OT secretion. There has also been a study on the effects of exogenous OT on social behavior in the African lion (*Panthera leo*), which has been the only study administrating OT to feline species^20^. In this study, after OT administration, African lions spent more time near their group members and were less wary of intruders outside the group. This suggests that exogenous OT affects social interaction in Felidae; however, knowledge of the effects of exogenous OT on inter-species relationship between cats and humans is still lacking.

The current study was designed following the protocols in a previous study^11^ investigating dog–human bonding mechanisms to elucidate the characteristics of the social behaviors between cats and humans. For comparative evaluation of the effects of OT between dog–human and cat–human social interactions, we analyzed the same behavioral index (i.e. gaze to human). We believe that this direct comparison could provide some important insights into the possible differences in mechanisms between dog–human and cat–human interspecies bonding. If OT-mediated social behaviors exist between cats and humans, as it does between dogs and humans, then certain effects would be expected. We predicted the following: (1) OT administration will increase cats’ social behaviors to owners (gaze, touch, vocalization, or proximity) compared with saline administration; and (2) sex differences in the effect of OT will be observed in cats as in the previous study where the effect was more conspicuous in female dogs than in males.

## Results

**Figure 1:**
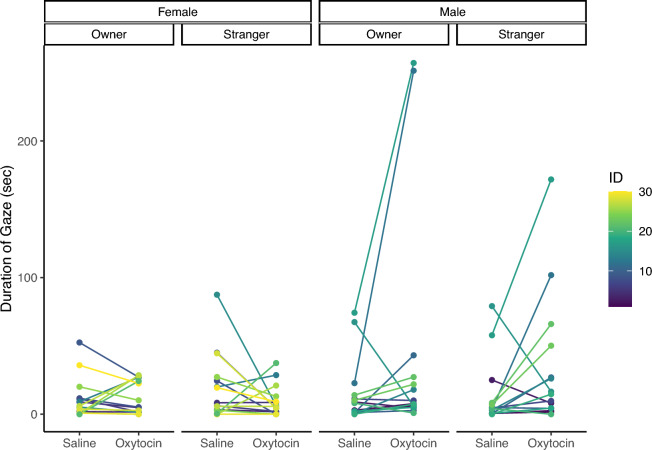
Individual duration of gaze following administration of oxytocin or saline.

**Figure 2:**
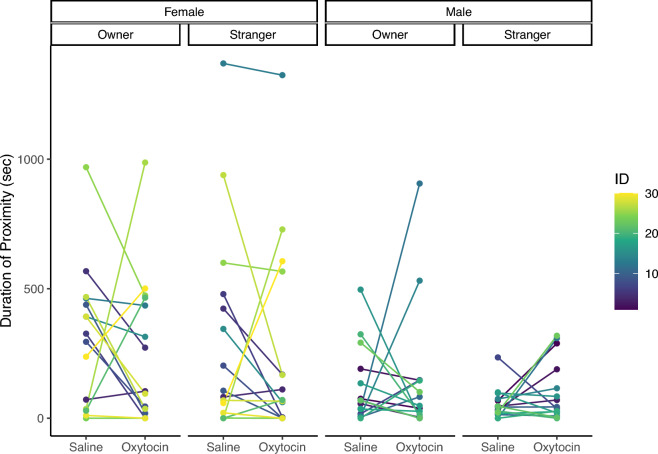
Individual duration of proximity following administration of oxytocin or saline.

We investigated cats’ social behaviors to two experimenters, the owner and a stranger, after nasal administration in a room of the owner’s house. Following our pre-registered methods of analyses, we conducted a linear mixed model (LMM) using cat sex, human experimenter (owner or stranger), and administration conditions (OT or saline) as explanatory variables for each dependent behavioral variable (gaze, touch, vocalization, and proximity). The main effects and interactions investigated are presented in Table 1. For gaze duration, there was no main effect of the experimenter (*χ*^2^ (1) = 0.112, *p* = 0.738) and condition (*χ*^2^ (1) = 3.615, *p* = 0.057). There was also no interaction between the experimenter and condition (*χ*^2^ (1) = 0.914, *p* = 0.339), but an interaction between condition and sex (*χ*^2^ (1) = 5.822, *p* = 0.016) was found (Fig. 1). A post hoc test with Bonferroni correction showed that the gaze duration was significantly longer in male cats administered OT than in those administered saline (*t*(83) = 3.054, *p* = 0.016; Table 2). For the duration of touch, there was no main effect of the experimenter (*χ*^2^ (1) = 0.394, *p* = 0.530) and condition (*χ*^2^ (1) = 0.077, *p* = 0.782). There was also no interaction between the experimenter and condition (*χ*^2^ (1) = 0.044, *p* = 0.835). For the number of vocalizations, there was no main effect of the experimenter (*χ*^2^ (1) = 1.616, *p* = 0.204) and condition (*χ*^2^ (1) = 0.361, *p* = 0.548). There was a main effect of the trial (*χ*^2^ (1) = 4.859, *p* = 0.028), with a higher number of vocalizations in the first trial than in the second. There was no interaction between the experimenter and condition (*χ*^2^ (1) = 0.038, *p* = 0.845). For the duration of proximity, there was no main effect of the experimenter (*χ*^2^ (1) = 0.394, *p* = 0.530) and condition (*χ*^2^ (1) = 0.077, *p* = 0.782). The main effect was observed for sex (*χ*^2^ (1) = 7.104, *p* = 0.008), indicating that females were closer to the experimenter than males (Fig. 2). There was no interaction between the experimenter and condition (*χ*^2^ (1) = 0.037, *p* = 0.848).

**Table 1:** Linear mixed model analysis results for the duration of gaze, duration of touch, number of vocalizations, and duration of proximity.

Response variable	Explanatory variable	χ^2^	*df*	*p*-value
Duration of gaze	Experimenter	0.112	1	0.738
	Condition	3.615	1	0.057
	Sex	1.654	1	0.198
	Trial	2.004	1	0.157
	Experimenter × condition	0.914	1	0.339
	Experimenter × sex	0.732	1	0.392
	**Condition** × **sex**	**5.822**	**1**	0.016*
	Experimenter × condition × sex	0.009	1	0.923
Duration of touch	Experimenter	0.001	1	0.978
	Condition	0.015	1	0.901
	Sex	3.340	1	0.068
	Trial	0.059	1	0.808
	Experimenter × condition	0.044	1	0.835
	Experimenter × sex	0.299	1	0.584
	Condition × sex	0.164	1	0.686
	Experimenter × condition × sexmale	0.377	1	0.539
Number of vocalizations	Experimenter	1.616	1	0.204
	Condition	0.361	1	0.548
	Sex	0.291	1	0.590
	**Trial**	**4.859**	**1**	0.028*
	Experimenter × condition	0.038	1	0.845
	Experimenter × sex	0.726	1	0.394
	Condition × sex	1.048	1	0.306
	Experimenter × condition: sexmale	0.282	1	0.596
Duration of proximity	Experimenter	0.394	1	0.530
	Condition	0.077	1	0.782
	**Sex**	**7.104**	**1**	0.008**
	Trial	1.247	1	0.264
	Experimenter × condition	0.037	1	0.848
	Experimenter × sex	0.536	1	0.464
	Condition × sex	0.893	1	0.345
	Experimenter × condition: sexmale	0.008	1	0.931

Significant values are in bold.

**p < 0.01, *p < 0.05.

**Table 2:** Results of post hoc analysis using the Bonferroni method for the interaction between administration condition and sex in the duration of gaze.

Data	Estimate	*SE*	*df*	*t*.ratio	*p*-value
Female_Oxytocin–Female_Saline	3.009	7.66	83.0	0.393	0.979
Female_Oxytocin–Male_Oxytocin	26.678	11.82	44.2	2.256	0.124
Female_Oxytocin–Male_Saline	3.296	11.77	43.5	0.280	0.992
Female_Saline–Male_Oxytocin	23.670	11.77	43.5	2.010	0.200
Female_Saline–Male_Saline	0.287	11.82	44.2	0.024	1.000
**Male_Oxytocin–Male_Saline**	**23.382**	**7.66**	**83.0**	**3.054**	0.016*

Significant values are in bold.

Furthermore, we conducted several exploratory analyses. First, we analyzed gaze duration using LMM with data only from cats whose owners were female to eliminate the possible confounding effect of the owner’s gender since most of the owners were females (28 out of 30) in our experiments. The results showed a main effect for condition (OT or saline) (*χ*^2^ (1) = 4.153, *p* = 0.042) and an interaction between condition and cat’s sex (*χ*^2^ (1) = 6.952 *p* = 0.008). Second, to examine the possibility that the male cats might have increased their gaze to general salient stimuli and not specifically to social stimuli, we analyzed using LMM the duration of gaze to a video camera placed on a tripod, a salient novel stimulus in the cats’ houses, in 15 male cats tested in the experiment. The results revealed no main effect of condition (*χ*^2^ (1) = 0.011, *p* = 0.917). In addition, we analyzed the effects of the duration of ownership between the owner and the cat on behavior toward the owner and the effects of OT. There was no correlation between the duration of ownership and the duration of gaze under OT (*r* = 0.09, *p* = 0.72) or saline (*r* = 0.28, *p* = 0.31) conditions.

## Discussion

In this study, we investigated the impact of exogenous OT on the social behaviors of cats, known for their solitary tendencies. The results indicate that intranasal administration of OT to cats resulted in an interaction effect between the OT/Saline condition and sex, with a significant increase in the gaze duration directed at humans observed in male cats. However, no changes were observed in other behaviors, such as duration of touch, number of vocalizations, and duration of proximity. Additionally, to control for the sex of the human participants, we conducted an exploratory analysis with cats owned by females. The analysis revealed a main effect of OT administration and an interaction effect between the OT/Saline condition and sex. This additional exploratory analysis supports the efficacy of OT administration.

The cats we studied showed an increase in gaze duration, which is thought to be due to the fact that intranasal administration of OT affects behavior through the OT neural system. Although historically known for its role in uterine contractions during childbirth and the secretion of breast milk, recent research has revealed OT’s impact on social behavior beyond reproduction and mother–offspring interactions. For instance, previous studies have suggested that OT plays a role in sociability, anxiety behaviors, peer recognition, trust, and the formation of bonds across species^21,22^. Studies on the effects of exogenous OT have shown that intranasal administration increases gaze directed toward the eye region in multiple species, including humans^9,23–25^. This may be related to a mechanism that enhances face processing by increasing attention to the eye region^26,27^. The action of exogenous OT on the nervous system, influencing the social behavior of gazing, suggests that similar effects were at play in the cats in our experiment.

We experimented on cats using a protocol similar to that used in prior research on dogs^11^. Our primary objective was to compare the effects of OT administration on interspecies social behaviors in terms of dog–human and cat–human interactions. Nagasawa et al. found that intranasal administration of OT in dogs increased the duration of gaze directed toward their owners. This increase in gaze was associated with a rise in urinary OT levels in the owners, suggesting a positive loop mediated by gaze and the OT neural system, similar to that observed between mothers and infants. Similarly, in our experiment with cats, we observed an increase in gaze duration, indicating OT’s impact on their social behavior. This study suggests that similar to its role in dog–human interactions, exogenous OT may also foster an affiliative relationship between species in cat–human interactions.

Contrary to our hypothesis, the effects of exogenous OT in cat–human interactions differed from those observed in dog–human interactions in two notable aspects. Firstly, in dogs, the increase in gaze duration was observed solely toward their owners, whereas in cats, there was no significant difference in gaze duration when distinguishing between owners and strangers. Secondly, while only female dogs exhibited an increased gaze following OT administration, this effect was exclusively observed in male cats. The causes of the differences between dogs and cats are discussed below.

The first difference could be attributed to the inherent social structures of cats and dogs. Dogs are group-oriented animals that distinctly differentiate between in-group and out-group members. Previous research has shown that gaze directed from dogs to their owners can enhance OT secretion in both parties as an attachment behavior^11^. This suggests a difference in gazing behavior toward owners (in-group) versus strangers (out-group). Although cats can distinguish between their owners and strangers, altering their behaviors accordingly^28^ and showing different responses to the voices of their owners compared to those of strangers^29^, studies using the Strange Situation Test (SST) have found no significant differences in cat behaviors toward their owners and strangers^30^. Considering that the ancestral species of domestic cats, the African wildcat, is solitary and does not form groups^12^, it is plausible that even domestic cats living with humans may not perceive their owners as in-group members. This hypothesis is supported by evidence that even cats with higher levels of OT exhibit infrequent affiliative behaviors, such as allogrooming, indicating that they do not consider other cohabiting cats as part of their in-group^31^. Therefore, cats may not distinguish between their owners and strangers in terms of in-groups and out-groups, resulting in no observable difference in their behavior toward both.

Another possibility is that the increase in gaze duration induced by OT administration was a response to general salient stimuli rather than a specific social stimulus. If this were the case, one might expect that the cats would also show an increased gaze duration toward a novel object, such as a camera mounted on a tripod, under the influence of OT. However, additional analysis on the gaze duration toward the camera revealed no significant differences between the OT and saline conditions, suggesting that the increased gaze duration is indicative of the influencing effect of exogenous OT on cats’ social behavior toward humans. Considering that the experiments were conducted during the COVID-19 pandemic, it is possible that cats could not differentiate between their owners and strangers due to mask-wearing. However, it is known that cats can recognize their owners’ voices^29^, and during the experiments, both the owners and strangers called the cat's name every four minutes, which was deemed sufficient for the cats to distinguish between them. Therefore, it is plausible that the cats differentiated between their owners and strangers but did not alter their gaze duration in response to either.

The second difference between cats and dogs, specifically the observation that exogenous OT increased gaze duration only in male cats, as opposed to female dogs, invites further consideration. Research across various animal species has revealed sex-specific effects of OT administration, which vary by species. Reports suggest that in males, OT may contribute to reduced vigilance or anxiety behaviors. For instance, in male mice, OT receptor response is linked to anxiety reduction^32,33^. In human males, intranasal OT administration has been found to decrease amygdala activity in response to social threats, thereby enhancing anti-anxiety effects^34^. The only previous study on OT administration in a felid species, the African lion, demonstrated reduced alertness or warning behaviors toward out-groups, although no gender differences were observed ^19^. At this stage, it is unclear why OT administration affected only female dogs and male cats. However, the increase in gaze duration observed in male cats could potentially be attributed to anxiety reduction fostering affiliative behavior. This study represents the first instance of OT administration in cats, and further verification across a variety of animal species is necessary to better understand these sex-specific effects.

Compared to those of the prior dog studies, the results of our cat experiments suggest that there may be distinct mechanisms underlying social behaviors in interactions between cats and humans. Dogs and cats are both domesticated companion animals that are closely involved in human life. However, the length of their domestication differs, with dogs living in human society much longer than cats (cats, ~ 10,000 years; dogs, ~ 15,000–100,000 years)^13–15^. Furthermore, as dogs have been historically used as collaborators in hunting activities^35^, their relationship with humans inevitably has a more cooperative nature. Meanwhile, although cats provide a service for humans in that they hunt mice, the relationship is less cooperative, and their breeding and feeding are not completely controlled by humans due to degrees of undomestication; therefore, the nature of communication between cats and humans may differ from that between humans and dogs. Dogs and cats have a similar ability to follow human pointing, but cats are less likely than dogs to gaze at their owners when faced with a problem-solving situation where they need support from their owners to get food^36^; that is, some behaviors of dogs and cats toward humans are similar, while others are different. Therefore, cats may have a different mechanism than dogs when doing social behaviors with humans.

Our experiment had several limitations. We focused primarily on comparing conditions between OT and saline administration without considering the social history or breed of the cats involved. It is possible that differences in how cats interact with humans, influenced by their interactions during the socialization period, could affect the response to exogenous OT. However, further analysis of the correlation between the rearing period of male cats and gaze duration under OT conditions revealed no significant correlation. This suggests that the observed differences between OT and saline are reflective of OT effects unrelated to the duration of human interaction. Previous studies indicate that interaction styles with humans can vary by breed^37^. However, in our study, 28 out of 30 cats were of mixed breed, indicating minimal breed-specific influence. Future research could investigate how breed or typical behavior toward humans affects the response to OT administration. Additionally, to administer OT/Saline without restraining the cats, they were given liquid treats during administration. Another possible limitation may be related to our administration method. However, this method of administration has been established in previous studies with several other animal species^9,38,39^, and we believe that it is also valid for cats. The fact that we observed some differences in the results between the OT and saline conditions proves that the administration itself was successful. Although we could not find any firm previous studies confirming the respiratory status of cats during feeding, we confirmed that the cats tested in our study breathed during the administration even while licking the liquid treats based on our video analyses. It is also possible that the intake of liquid treats during the administration enhanced OT secretion. However, it is unlikely that this had an impact on the results, especially on the observed differences between the OT and saline conditions, as the amount of their intake was minimal, and the same treats were provided in both conditions. We did not observe any apparent difference in cats’ intake during administration between the OT and saline conditions, although we did not record the exact amount of intake, and thus we cannot comment on the possibility of OT secretion caused by treat intake. Previous studies applying a similar method of administration while feeding have not reported any significant impact of this method on the results^9,20^.

To the best of our knowledge, this study represents the first exploration of OT administration in the context of interspecies social relationships involving humans and cats. We found both similarities and differences in the effects of exogeneous OT on interspecific social relationships in terms of dog–human and cat–human interactions. We believe that this research lays the foundation for further comparative psycho-endocrinological studies on the mechanisms underlying interspecies bonding. This study provides practical implications for enhancing animal welfare and fostering harmonious relationships between humans and companion animals. While we anticipate the need for larger sample sizes in future studies involving cats, the suggested benefits of exogenous OT in reducing anxiety during interspecies interactions hold promise for the field of animal husbandry. In this regard, our newly developed non-invasive method of OT administration can be applied to daily treatments for cats and other companion animals facing challenges in their relationships and interactions with humans. We aspire that this study will contribute to the betterment of companion animal welfare within human society, thereby enhancing the overall well-being of humans themselves.

## Methods

We conducted this study using the method described in the preregistration (https://osf.io/d3wkh/).

### Participants

The experiment was conducted in the owner's houses in Japan from November 2021 to August 2022. It is recognized that cats require a period of adjustment to new environments, unlike dogs^38^. Therefore, we conducted the experiment at the owner’s home to observe the cats' natural behavior. We tested each cat in two trials, separated by an interval of more than one month. The cats in this experiment were individuals who did not hide when the experimenter entered the room, and who were not averse to the OT administration device. We did not conduct any tests to familiarize the cats with the device. The amount of interaction between the cats and their owners before the experiment was not controlled. The provision of food and water was not regulated. The cats and their owners were together in the house before the experiment. The cat owner was paid 1000 yen for their experiment in each trial. The owners were recruited through social networking platforms such as Twitter and Instagram. Informed consent was obtained from all participants for both study participation AND publication of identifying information/images in an online open-access publication. Seventy cats (31 males and 39 females, all cats were neutered) were experimented in the first trial, whereas only 30 cats (15 males and 15 females) completed the second trial. The reasons for dropout were as follows: two males because the owner was infected with coronavirus at the time of the second trial; one male because the cat started taking medication due to illness before the second trial; and 13 males and 24 females refused to accept OT administration for 1 min through a nebulizer mask. The 30 cats included in both trials ranged in age from 1 to 12 years (mean: 5.69 ± SD 3.34, median: 5.08); their breeds were as follows: 28 mixed breeds, 1 British Shorthair, and 1 American Shorthair. All cats were neutered/spayed. The owners were 2 males and 28 females aged 29–75 years (mean: 46.16 ± SD 9.73, median: 44; Supplementary Table S1). The same female experimenter (MH) performed all tests and wore the same clothing and equipment.

### OT administration

**Figure 3:**
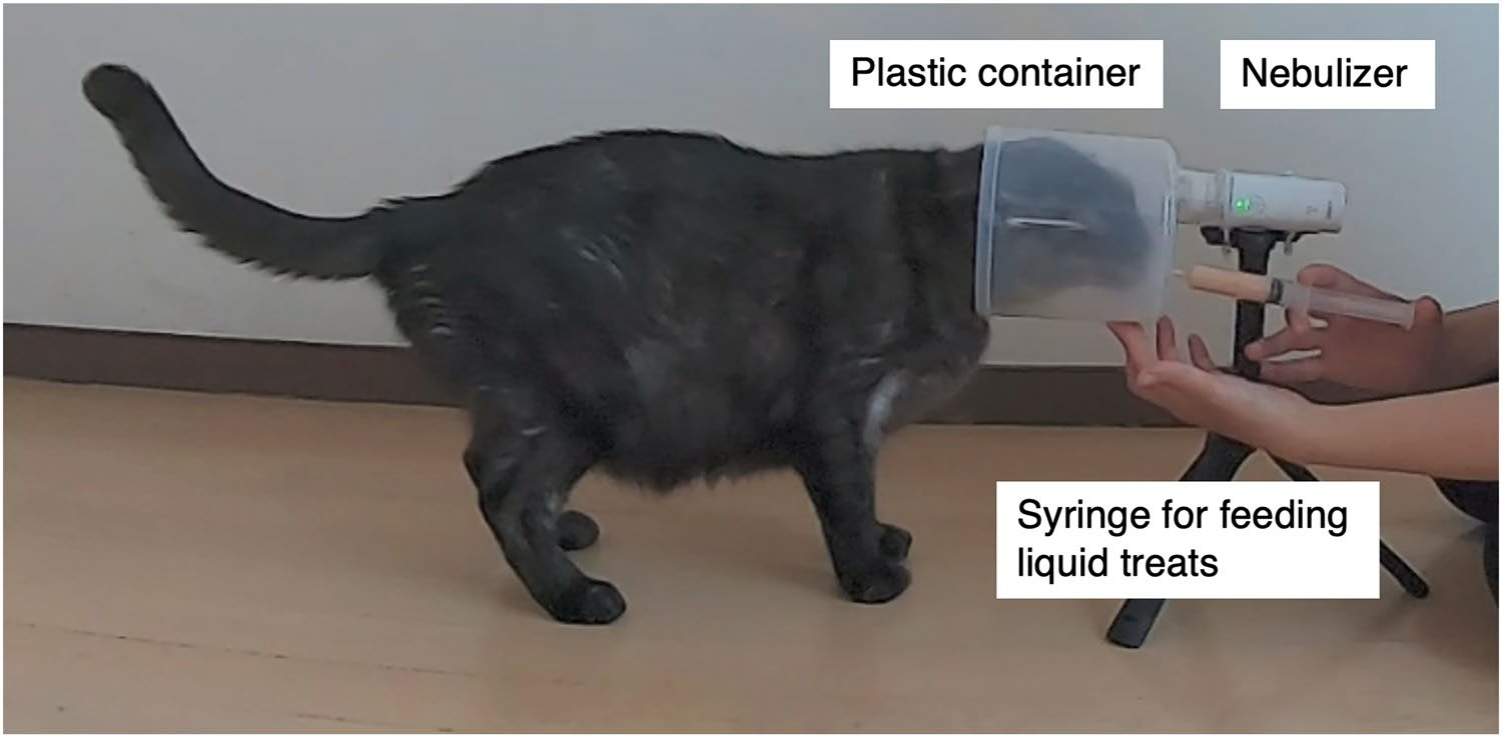
Method of administering oxytocin or saline to cats using a nebulizer.

The experimenter used a portable nebulizer (NE-U22-4; Omron, Kyoto, Japan) with a plastic container (13 cm diameter × 12.5 cm depth) and administered OT (4084-v; Peptide Institute, Inc., Osaka, Japan) or saline (Otsuka Normal Saline; Otsuka Pharmaceutical, Tokyo, Japan) while feeding the liquid treats (CIAO Churu, Tsuna; Inaba Petfood Corporation, Shizuoka, Japan) using a syringe. Ingredients of the liquid treats were water, tuna, tapioca starch, natural flavors, guar gum, natural tuna flavor, vitamin E supplement, taurine, and green tea extract. The administration time was 1 min, measured using a stopwatch (CASIO HS-3C). If the cat took its face out of the container during administration, the stopwatch was temporarily stopped, and the measurement was resumed when the cat returned its face into the container. Administration was stopped when the total time for the cat took 1 min to put its face in the container (Fig. 3). OT was administered at 160 IU/mL and nebulized at a rate of 0.25 mL/min; this means that approximately 40 IU of OT was nebulized during the period. The dosage of 40 IU administered in this study falls within the range established by a previous research on dogs^11^ and was selected in consideration of the potential for leakage from the container during administration. The administration method using portable nebulizer was newly customized based on the previous method developed for captive chimpanzees and bonobos ^9^.

**Figure 4:**
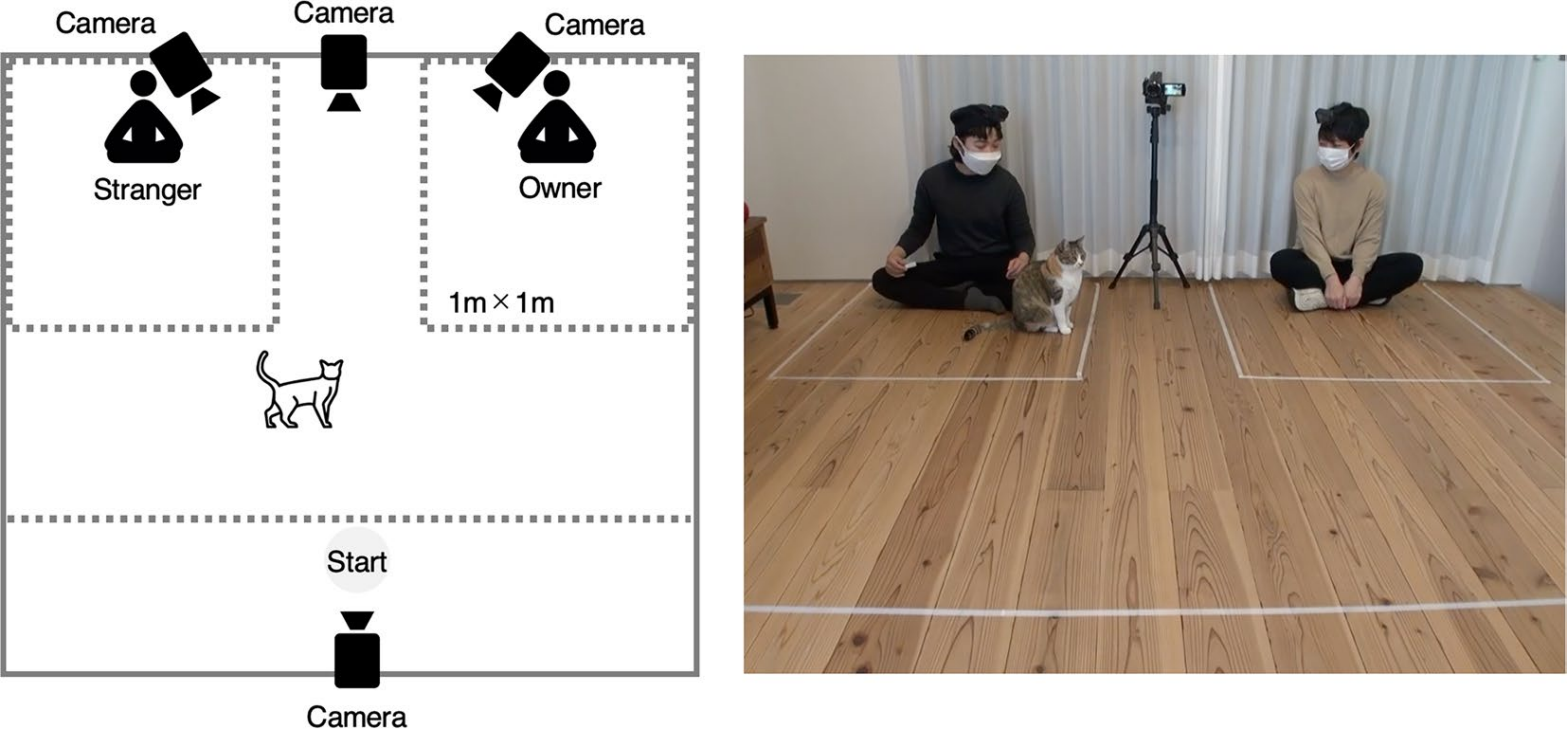
Experimental setup at the owners' homes. The owner and stranger wearing a head camera sat in a 1 m × 1 m square.

We waited for 10 min after administration and recorded the cats’ behavior for the next 32 min using a video camera. Two experimenters (a cat owner and a stranger) wore head cameras (GoPro HERO7 and HERO9). The experimenter installed video cameras (Sony HDR-CX700v and HDR-CX670) on tripods at two locations in the room. The owner and experimenter sat in a 1 m × 1 m frame taped off for marking, and the owner placed the cat 2 m outside to start the experiment (Fig. 4). We did not restrain cats trying to leave the observation area, and we measured their behavior while in the observation area. The owner and experimenter stared at the cat's eyes throughout the experiment. They called the cat’s name once every 4 min and swapped the sitting area of the owner and experimenter every 8 min to control the influence of location preference. When the cat sat on the owner's or experimenter's lap, the person whose lap the cat sat on returned the cat to the starting position while changing places. The owners and experimenters were blinded to whether the cats were administered OT or saline. Each cat was administered OT and saline in a counterbalanced order. All experimenters wore surgical masks during the experiment, given the restrictions implemented during the COVID-19 pandemic.

### Behavior analysis

Using the analysis software ELAN (version 6.4; Max Planck Institute for Psycholinguistics, The Language Archive, Nijmegen, The Netherlands; https://archive.mpi.nl/tla/elan), the following were counted (Supplementary Table S2): duration of gaze, the time the cat looked at the owner or stranger; duration of touch, the time the cat made contact with the owner or stranger, such as licking, touching, or rubbing; number of vocalizations, the number of times the cat purred with its face toward the owner or stranger, not including the number of times it purred toward the outside the room; and duration of the proximity to owners and strangers, wherein proximity was the time when three of the four legs of the cat are within a 1 m square area of the owner or stranger. The video data were coded by a coder without knowing whether the cats were administered OT or saline. To evaluate the coding validity, the videos of eight cats (26% of the 30 cats) were coded by a second coder, and we confirmed the inter-coder reliabilities were sufficiently high (duration of gaze *r* = 0.832, *p* < 0.001; duration of touch* r* = 0.923, *p* < 0.001; number of vocalizations *r* = 0.905, *p* < 0.001; and duration of proximity *r* = 0.996, *p* < 0.001).

### Statistical analysis

As written in preregistration, we performed a linear mixed models (LMM) using the 'lme4' package in R (version 4.2.2). The explanatory variables were the conditions (OT/saline), sex of the cat (male/female), experimenter (owner/stranger), and the interaction of all these effects. We also included the trials (1st or 2nd) as fixed control factors in the model. Response variables were behavioral measures (duration of gaze, duration of touch, number of vocalizations, and duration of proximity) of the cat toward the owner or the stranger (experimenter). The random effect was experimenter ID. The α-level was set to 0.05. As an additional exploratory analysis, we conducted the same LMM on a dataset that included only female pet owners. Similarly, we analyzed the duration of time the cats looked at a camera mounted on a tripod using the same LMM approach. To analyze the effects of the cat's age and the duration of cat ownership on their behavior toward their owners and the effects of OT, we conducted correlation analyses between age and duration of gaze, as well as between the duration of ownership and duration of gaze (Supplementary Table S3).

### Ethics statements

This study adhered to the ethical guidelines of Kyoto University which follows “Guidelines for Proper Conduct of Animal Experiments”, and was approved by Wildlife Research Center, Kyoto University (WRC-2022-017A). Informed consent was obtained from all owners. This study was carried out in compliance with the ARRIVE guidelines. All procedures adhered to the Japanese Act on the Welfare and Management of Animals.

### Supplementary Information

Supplementary Tables.

## Data Availability

The data supporting this article are included in Supplementary Electronic Information.
